# Wastewater Treatment Using a Photoelectrochemical Oxidation Process for the Coffee Processing Industry Optimization of Chemical Oxygen Demand (COD) Removal Using Response Surface Methodology

**DOI:** 10.1155/2022/1734411

**Published:** 2022-07-31

**Authors:** Firomsa Bidira Abdi, Zerihun Asmelash Samuel, Seifu Kebede Debela, Temesgen Abeto Amibo

**Affiliations:** ^1^Faculty of Civil and Environmental Engineering, Jimma Institute of Technology, Jimma University, Jimma, P.O. Box 378, Ethiopia; ^2^School of Chemical Engineering, Jimma Institute of Technology, Jimma University, Jimma, P.O. Box 378, Ethiopia

## Abstract

The elimination of organic compounds in coffee processing effluent utilizing electrochemical oxidation (ECO) as well as a combination of electrochemical oxidation (ECO) and ultraviolet and hydrogen peroxide (UV/H_2_O_2_) was explored. Then, the percentage reduction of chemical oxygen demand (COD) was investigated. The effect of different experimental factors such as solution pH, sodium chloride (NaCl) concentration, calcium chloride (CaCl_2_) concentration, electric current, electrolysis duration, and hydrogen peroxide dosage on the percent removal efficiency of the hybrid electrochemical oxidation (ECO) with the ultraviolet and hydrogen peroxide (UV/H_2_O_2_) process has been investigated. The response surface methodology (RSM) based on central composite design (CCD) was used to organize the trial runs and optimize the results. The hybrid electrochemical oxidation (ECO) with the ultraviolet and hydrogen peroxide (UV/H_2_O_2_) process removed 99.61% of the chemical oxygen demand (COD) with a low power usage of 1.12 kWh/m^3^ compared to the other procedures, according to the experimental data analysis. These findings were obtained with a pH of 7, a current of 0.40 A, 1.5 g of CaCl_2_, and a total electrolysis period of 40 minutes. When it came to eliminating organic compounds from coffee manufacturing effluent, CaCl_2_ outperformed NaCl. Analysis of variance (ANOVA) with 95% confidence limits was used to examine the significance of independent variables and their interactions.

## 1. Introduction

Wastewater treatment is becoming an increasingly important aspect of industrial activities and contamination from such activities affects the environment. Proper treatment is essential before this wastewater may be discharged into water bodies; otherwise, the environment and human life may be harmed [1, 2]. Electrochemical oxidation for wastewater treatment has been studied since the nineteenth century. Over the last three decades, research has concentrated on the oxidation efficiency and electrochemical stability of electrode materials, variables influencing process performance, and the investigation of pollutant degradation processes and kinetics [3]. Electrochemical oxidation has been identified as one of the most successful strategies for pollutant degradation in textile wastewater, landfill leachate, simulated wastewater, olive mill wastewater, paper mill wastewater, and industrial paint effluent [4]. Several innovative technologies have been developed and tested in recent decades, with improved oxidation processes being among the most promising. The OH radicals can also alter the chemical structure of resistant organic molecules, transforming them into simpler compounds with reduced molecular mass and less biodegradable toxicity to microbes. Hydrogen peroxide (H_2_O_2_) has been used for many years to remove organic materials from industrial wastewater and purify wastewater [5, 6]. Because the hydroxyl radical (OH) has a limited lifetime due to its instability, it must be created in situ continually via chemical or photochemical processes to complete the oxidation process.

Currently, the development of various sectors contributes to the economic expansion of countries, but our environment is contaminated owing to a lack of adequate management of wastewater created from various processing units of the industries [7–9]. For example, coffee is one of the backbones of the Ethiopian economy, but due to a lack of technology for treating coffee processing wastewater, water sources were polluted and various useful organisms were harmed, particularly due to coffee processing wastewater can create bad odors and sludges in water bodies. To address these issues, the approach was to create low cost, simply deployable, and environmentally acceptable technologies such as enhancement of photoelectrochemical oxidation [10, 11].

The addition of H_2_O_2_ to ECO enhanced the removal efficiency of contaminants from wastewater. Many sophisticated oxidation processes were previously performed using electrochemical oxidations, but today, it is feasible to boost removal efficiency from coffee processing effluent and other wastewater by combining ECO with UV/H_2_O_2_. Photoelectrochemical treatment was simply adaptable and could be completed at a low cost and in a short amount of time; also, photoelectrochemicals are often simpler, cleaner, less costly, and more efficient than traditional and chemical AOPs [12, 13]. Previously conducted studies relied solely on electrochemical wastewater treatment or coupled with UV/H_2_O_2_ treatment. Organics compounds can be treated with H_2_O_2_ to lower their toxicity and enhance their biodegradability [1]. Based on prior information, this study integrated both technologies and enhanced the removal efficiency of effluent from the coffee processing sector. The ECO/UV/H_2_O_2_ coupled technology may make a significant contribution to this problem, since it can be employed in several ways as selective separation technology, degrading processes and enabling chemical-free wastewater treatment. Furthermore, there has been a significant surge in new advances in electrochemical processes to treat wastewater released from industries [14]. Second, many studies on wastewater from cafeterias, industries, and pharmaceuticals have been conducted in different studies previously, but this study is focused on coffee processing wastewater, with the primary dependent variable being chemical oxygen demand. The primary goal of this research is to look into the photoelectrochemical oxidation technique for coffee processing wastewater treatment. The purpose of this study is to investigate the efficiency of a photoelectrochemical oxidation process for the removal of COD from coffee processing wastewater and to determine the effect of various experimental parameters such as reaction time, type of electrolyte, pH, current, and UV/H_2_O_2_ on removal efficiency. To statistically optimize the parameters, response surface methodology (RSM) was employed. The CCD model was used to optimize the findings from RSM, since it is more efficient than Box-Behnken Design (BBD) and is also outside of the design space box created by the factorial section of the design. The dependent variable optimized in this study was COD, while the independent variables were electrolyte concentration, time, pH, UV/H_2_O_2_, and current, which were regulating factors that determined the removal capacity of this approach.

This study focused on wastewater treatment in the coffee processing industry, and the impacts of key parameters such as electrolyte concentrations, time, pH, UV/H_2_O_2_, and current were explored. The novelty of this study is that it compares the performance of different types of electrolytes and electrolyte concentrations on COD removal. Furthermore, the effects of UV on COD removal, as well as UV combined with hydrogen peroxide (UV/H_2_O_2_), were considerable. The novel discovery in this study is that the effects of electrolytes are dependent on the ions that carry electrons, and the optimization is based on this concept.

## 2. Materials and Methods

The following equipment was used in this study: beaker, magnetic stirrer (model: HSC-19T, SN:201802307), desiccator, oven-dry (model number: DHG-9203A), filter paper, COD reactor (model number: JGR.01), COD kit, conductivity meter, electrode (Fe, Al, and steel), DC-power supply, UV-spectrophotometer (make and model: PerkinElmer Lambda 25), vacuum pump, vacuum hood, multimeter, heaters, conical flasks, pH meter (model number: PH-920), standard flasks, and Erlenmeyer flasks, measuring. Chemicals used for experimentation were mercury sulfate (HgSO_4_), ferrous ammonium sulfate (Fe (NH_3_) SO_4_), silver sulfate (Ag_2_SO_4_), ferroin indicator (Fe(o-phen)_3_SO_4_), potassium dichromate (K_2_Cr_2_O_7_), and sulfuric acid (H_2_SO_4_) were used for COD; hydrogen peroxide (H_2_O_2_) was used as an oxidizing agent and supporting reagent.

### 2.1. Sample Collection and Preservation Method

Wastewater was collected in plastic jar cans from the Jimma zone, Yabbu town coffee processing wastewater disposal point for three days. During the research period, about 200 liters of wastewater were collected. After soaking the jar cans in 10% HCl for 24 hours, they were carefully cleaned and washed with distilled water; this is to remove dirty materials from the jar. During transit to the laboratory, plastic bottles holding samples were packed in a box. Plastic boxes were used to shield samples from the sun and keep them at a constant temperature of room temperature during travel. The sample is transferred to the laboratory by the sample preservation for characterization protocol.

The sample was produced for evaluation of the UV/H_2_O_2_ and electrochemical oxidation processes individually and in combination. The sample was conserved by maintaining it at its highest holding temperature until the start of each parameter's laboratory measurement process. The maximum holding duration is maintained and carried out by the WHO/UNEP standard methodology and water treatment laboratory guides from 2004.

### 2.2. Experimental Setup

**Figure 1:**
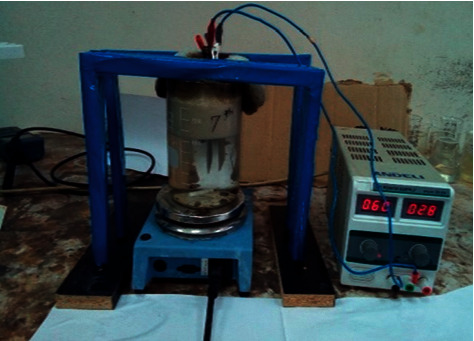
Experimental setup of the treatment method.

An electrochemical reactor and a UV lamp make up the experimental setup for the hybrid UV/H_2_O_2_ oxidation process. The COD content of the wastewater is determined. Through the perforations in the rubber stopper, the working electrode, reference and counter electrodes (Fe, Al, and steel), and the pipette (for bubbling) are introduced. As shown in Figure 1, the experimental setup was conducted to treat wastewater in the coffee processing industry.

### 2.3. Analysis Methods

Following the successful completion of the data collection, the data were processed and analyzed using response surface methodology (RSM). It was optimized and analyzed utilizing both qualitative and quantitative data analysis methodologies. All of the data were compared to WHO guideline limits (2004).

#### 2.3.1. Removal Efficiency

The COD test is an indicator of organic components in wastewater, and it is estimated using equation (1) to determine the percentage removal of COD. Additionally, equations (2) and (3) were used for the determination of COD found in wastewater. The process's performance was assessed based on COD responses, removal efficiencies, and power usage. RSM was a type of environmental modeling that was used to analyze laboratory data using empirical formulas.

(1) $$\begin{array}{c} \%\text{COD removal}=\frac{{\text{COD}}_{i}-{\text{COD}}_{O}}{{\text{COD}}_{i}}*100, \end{array}$$

where COD_i_ and COD_t_ are the chemical oxygen demands in mg/l at initial (*t* = 0) and at any reaction time (*t*), respectively [15].

The COD (mg/L) of each run was calculated using the following formula, which was primarily utilized to calculate the quantity of COD present in the solution [6].

(2) $$\begin{array}{c} \text{COD}=\frac{\left(A-B\right)}{\text{The volume of sample}}*N*8*1000, \end{array}$$

where *A* is the volume of FAS for blank, *B* is the sample, and volume of sample = 2.5 mL.

(3) $$\begin{array}{c} \text{Normality of FAS }\left(N\right)=\frac{\text{Weight of FAS used in FAS solution preparation}}{\text{Equivalent weight of FAS}}. \end{array}$$

#### 2.3.2. Response Surface Methodology

The response surface methodology approach is a collection of strategies for experimenting to find the best operating conditions. Typically, this entails conducting numerous experiments and utilizing the results of one experiment to guide the next [16]. The following parameters were varied in this study: pH (5–9), electrolytic concentration (0.5–2.5 g) of NaCl/CaCl_2_, current (0.2–0.6 A), reaction duration (20–50 minutes), and H_2_O_2_ concentration (2–5 ml) in 200 ml of distilled water. These parameters were selected based on experimental results, which imply that the experiments were done by adjusting one parameter at a time while keeping the other variables constant, which aids in determining the maximum adsorption value on a single variable. As a result, as given in Table 1, these inputs provide the number of experimental runs, PH range, duration, current, and electrolyte created by using RSM based-optimization. As a result, as given in Table 1, these inputs provide a range of pH, time, current, and electrolyte that was developed using RSM software with several experimental runs. In all, the total numbers of experiments performed were 156 trials from these sixty for electrochemical oxidation with NaCl/CaCl_2_ and 96 trials for a combination of electrochemical oxidation with NaCl/CaCl_2_ and UV/H_2_O_2_) were done in the laboratory utilizing an aluminum electrode and electrode spacing is 1 cm. The CCD model with four components was used in this study to improve the adsorption efficiency of parameters. The experimental design was based on five-level experiments. The total number of experiments (*N*) may be computed as follows.

(4) $$\begin{array}{c} Y_{i}=\beta_{0}+\sum_{i=1}^{4}\beta_{i}\cdot X_{i}+\sum_{i\leq j}^{4}\sum_{j}^{4}\beta_{ij}\cdot X_{i}\cdot X_{j}+\sum_{i=1}^{4}\beta_{ii}\cdot X_{i}^{2}+e, \end{array}$$

where *Y*_*i*_ is the response variable, *β*_0_ is the model (regression) constant, *β*_*i*_ is the linear terms, *β*_*ii*_ are the squared terms (second order), *β*_*ij*_ are the interaction terms, *X*_*i*_ and *X*_*j*_ are the independent variables, *e* is a random error, and *k* = 4 is the number of parameters.

## 3. Results and Discussion

### 3.1. Removal Efficiency of Electrochemical Oxidation Using NaCl and CaCl_2_

Depending on the findings, the color is extremely dark with 2.95 abs, the temperature is 43°C, and the COD is 7680 mg/L. NaCl is an electrolyte that is used to improve conductivity and decrease the amount of voltage provided to wastewater during the treatment process by producing Na^+^ and Cl^−1^. Calcium chloride (CaCl_2_) is an electrolyte that is used to improve conductivity and lower the amount of voltage provided to the wastewater during the treatment process to boost removal efficiency by producing Ca^+2^ and Cl^−1^. The experiments were repeated three times, with the average value used for analysis. From Table S3, the results for standard deviation are available based on COD removals with NaCl, COD removals with CaCl_2_, COD removals with a combination of NaCl and UV/H_2_O_2_, and COD removals with a combination of CaCl_2_ and UV/H_2_O_2_ varied from the arithmetic mean by 0.6214, 0.3239, 0.9337, and 0.9876, respectively. This figure implies that the standard deviation value is less than one, indicating that the variance between experiments is low.

### 3.2. Removal Efficiency of Electrochemical Oxidation Using NaCl and CaCl_2_ Combination with UV/H_2_O_2_

At this step, a combination of an electrolyte NaCl with ultraviolet light and hydrogen peroxide (UV/H_2_O_2_) is required to maximize the reduction of hydroxyl ions and the effectiveness of pollutant removal. Similarly, a combination of an electrolyte CaCl_2_ with ultraviolet light and hydrogen peroxide (UV/H_2_O_2_) is required to boost the minimization of hydroxyl ions and the effectiveness of pollutant removal as given in Table 2.

### 3.3. Effect of Operating Parameters on COD Removal Efficiency

The factors that have a significant impact on the EO and US/EO processes, such as solution pH, electrolyte concentration (NaCl/CaCl_2_), electric current, and reaction duration, were investigated in terms of COD removal to power consumption. The effects of the electrode were obvious in that COD and ammonia concurrently by indirect oxidation during the electrolysis process [17].

#### 3.3.1. The Effects of pH

**Figure 2:**
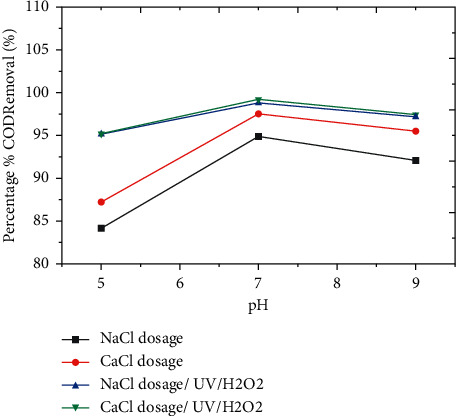
Effect of pH on removal efficiency COD from coffee processing wastewater.

The phrase pH effects are used to convey the strength of a liquid's acid or alkaline state. Higher alkalinity waters have higher pH, and in the EO and UV/H_2_O_2_ processes, the pH value of the solution is critical in the removal of pollutants from wastewater. To evaluate the influence of pH on process performance, it is altered in the range of 5–9 by adding the drop of either NaOH or H_2_SO_4_ in a solution. The neutral circumstances appear to be more promising for reducing COD elimination. Because more oxidant is created in the neutral medium, it decreases in the basic medium due to a considerable drop in the redox potentials of H^+1^ with decreasing pH. The best COD removals could be obtained in the neutral pH value with aluminum electrodes [17]. The effect of pH on COD removal potency is shown in Figure 2. The best COD elimination was found using aluminum electrodes at neutral pH [17]. The impact of pH on COD percent removal potency is shown in Figure 2.

#### 3.3.2. Effect of Electrolysis Time

**Figure 3:**
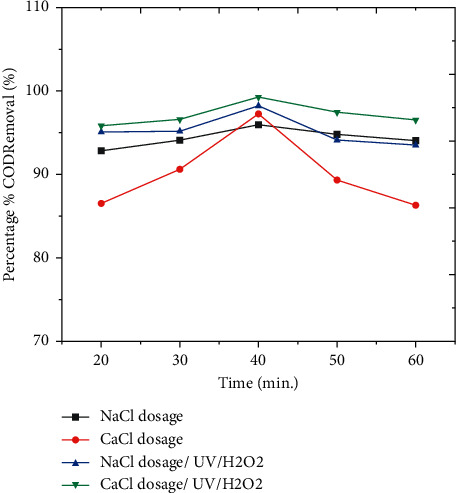
Effect of time on COD removal efficiency from coffee processing wastewater.

From Figure 3, the structure of the sludge may vary over time, affecting pollutant removal effectiveness as well as the settling ability and float ability features of the flocs. According to the research, longer reaction periods result in lower removal percentages, which might be attributed to metal hydroxide sequestration at the electrode surfaces [18]. When it comes to wastewater treatment in the coffee industry, both lengthy and short treatment times have low removal efficiency. According to this study, the optimal removal of COD was obtained at a time of 40 minutes.

#### 3.3.3. Effect of Electric Current

**Figure 4:**
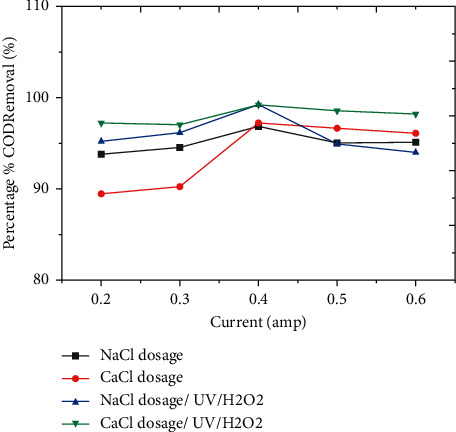
Effect of current on COD removal efficiency from coffee processing wastewater.

In reality, the current is proportional to voltage. There was an increase in aluminum dissolution in the solution; hence, the current increased. As a result, the generation of hydroxide Al (OH)_3_ is accelerated. When a greater voltage is applied, oxygen evolution occurs, resulting in a decrease in the efficiency of organic oxidation [19]. The oxidation of poisoning products is generated at the anode surface when the process is carried out at higher voltages. Coffee manufacturing wastewater treatment is unaffected by extremely high current as shown in Figure 4.

To improve the conductivity of the wastewater to be treated, table salt was commonly used. Chloride ions were discovered to greatly diminish the negative effects of other anions such as HCO^3−^ and SO4^2−^, in addition to their ionic involvement in transporting the electric charge. The presence of carbonate or sulfate ions causes the precipitation of Ca^2+^ or Mg^2+^ ions on the electrodes' surfaces and forms an insulating layer. Energy usage is reduced when electrolyte concentration rises. In the AO and AO-H_2_O_2_ processes, organic compound elimination and mineralization occur more quickly in the presence of NaCl or CaCl_2_ [20]. The voltage between electrodes would be substantially increased by this insulating layer, and it results in a considerable reduction in current efficiency. Therefore, the increase in conductivity happened due to the addition of NaCl and CaCl_2_, and at the same time, power consumption was reduced. Furthermore, chlorine produced electrochemically has been demonstrated to be useful in water disinfection [21]. As a result, for the tests, a concentration of 0.5–2.5 g/L NaCl and CaCl_2_ was used. When compared to the other components, the amount of electrolyte factor has a bigger impact on reaction. This is because NaCl/CaCl_2_ raises the conductivity of the EO system, which improves the removal of percent of COD. Due to the increase in ions from +1 (NaCl) to +2 (CaCl_2_), CaCl_2_ produces a more efficient outcome than NaCl/CaCl_2_. The addition of a supporting electrolyte (NaCl or CaCl_2_) was done to boost the solution's conductivity [22].

#### 3.3.4. Effect of Electrolyte Solution

**Figure 5:**
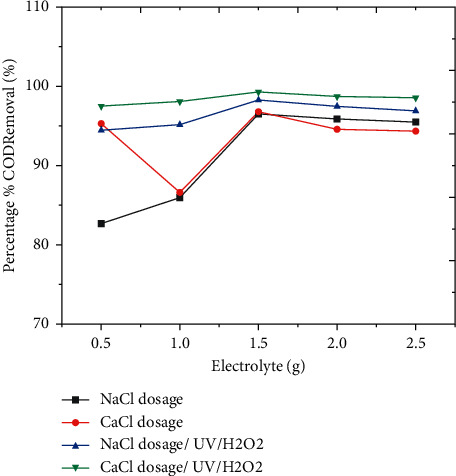
Effect of electrolyte concentration on COD removal efficiency by using NaCl.

As shown in Figure 5, the greatest percent of COD elimination with NaCl was 95.2%, whereas CaCl_2_ was 96.4%. By including an appropriate amount of electrolyte, the conductivity of the solutions was adjusted to the correct values. Secondary reactions, such as direct oxidation of organic molecules and Cl ions present in wastewater, may occur if the anode potential is sufficiently high. Within the powerful advanced oxidation technologies, indirect electro-oxidation processes represent a viable alternative for the destruction of high molecular weight substances. In particular, the elimination of COD makes it a promising technology for the treatment of high conductivity wastewaters [23].

Strong oxidants, such as active chlorine species, destroy organic load in these processes (ACS). An electron transfer to the anode reaction 1 (*R*_1_) generates ACS from chloride in water, which interacts with water to produce a hypochlorous acid reaction (*R*_2_). The equilibrium between hypochlorous acid and hypochlorite ion in water is dependent on the concentration and pH of the solution, according to the speciation of chlorine in the water. Reaction number 3 (*R*_3_) is aside from these active species; the chloride radical is produced by anode direct oxidation (*R*_4_). As a result, the chlorine gas produced can oxidize contaminants. The energy consumption of aluminum is larger, while the consumption of electrodes is lower. Higher conductivity appears to promote high process efficiency.

(5) $$\begin{array}{c} 2{\text{Cl}}^{-}⟶{\text{Cl}}_{2{\left(\text{aq}\right)}^{+}}\left(2e^{-}\right)\left(R_{1}\right) \end{array}$$

(6) $$\begin{array}{c} {\text{Cl}}_{2}+{\text{H}}_{2}\text{O}⟶\text{HClO}+{\text{Cl}}^{-}+{\text{H}}^{+}\left(R_{2}\right) \end{array}$$

(7) $$\begin{array}{c} \text{HClO}⟷{\text{ClO}}^{-}+{\text{H}}^{+}\left(R_{3}\right) \end{array}$$

(8) $$\begin{array}{c} {\text{Cl}}^{-}⟶\text{Cl}+e^{-}\left(R_{4}\right) \end{array}$$

The percentage of COD removed rise with an increase in electrolyte dose of g/L, but the highest amount of electrolyte did not affect removal efficiency. The oxidation of the organic component occurred immediately on the electrode surface, by increasing the current (amp).

#### 3.3.5. Effect of Ultraviolet Light/Hydrogen Peroxide (UV/H_2_O_2_)

**Figure 6:**
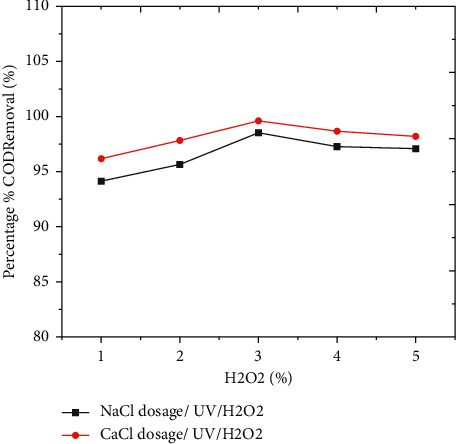
Effect of hydrogen peroxide on COD removal efficiency by using UV/H_2_O_2_ and NaCl.

From Figure 6, these coupled technologies might create synergistic effects for the removal of organic matter; ECO performance could be increased by pairing it with hydrogen peroxide (H_2_O_2_) and UVC light [24]. The elimination of organic contaminants contained in water from an advanced primary treatment (APT) was accomplished using both a batch photoreactor and systems that processed samples with UV light and H_2_O_2_ variables [25].

A photoelectrochemical reactor with a maximum capacity of 1 L of coffee processing wastewater and a UV light were utilized in the photoelectrochemical tests (model PUV-1022 Heraeus). It has a 40 cm length, an emission spectrum ranging from 200 to 460 nm, 60 Watts, 220 Volts, and 11.4 amps of current. A demineralized coffee manufacturing waste was used in the photoreactor. As a result, as the dosage of hydrogen peroxide grows the removal efficiency of organic substances and color increases as well, but once the optimal dose was achieved, there is no further improvement in removal efficiency.

### 3.4. Optimization by Response Surface Methodology

In this study, electrochemical parameters were statistically optimized using RSM, as given in Table 3. RSM is a type of regression analysis that uses the controlled values of the independent variables to predict the value of a dependent variable. RSM was used to optimize an experimental parameter for a different process; it is used in the advanced oxidation process. It is a highly efficient procedure because it not only finds the optimum operating conditions to maximize a system's performance but also generates a response surface model that predicts a response based on a combination of factor levels and response [26–29]. It also shows the relative amplitude and impact of various factors on the response, as well as their interactions. They have been used to mimic a wide range of wastewater treatment systems and processes [30–33]. All laboratory findings are given in Tables 1–3, which included influencing parameters and color absorbance at 450 nm wavelengths for the removal efficiency of COD. The major goal of this research was to find the best-operating parameters for an efficient treatment of coffee manufacturing wastewater. The following are the findings of the studies in terms of COD removal rate and power usage for ECO and UV/H_2_O_2_. Estimation models were used to improve the replies for establishing optimal spots for operating circumstances and obtaining the greatest removal efficiency [34–37]. COD removal was set to its maximum value to obtain the best removal performance under operational conditions. At the parameters maintained to pH of 7, the reaction time of 40 minutes, an electric current of 0.4 amperes, and a salt concentration of 1.5 g/L were the best conditions for independent variables. The model's degree of desirability was equal to 1 under these conditions. Tables S1 and S2 provide information on the analysis of the variance test; if the *p* value is less than 0.05, the parameter is significant; if the *p* value is greater than 0.05, the parameter is insignificant, which implies it does not affect the response.

Many parameters influence the effectiveness of photoelectrochemical oxidation, including pH, electrolyte concentration, electrolysis duration, current density, and turbulence. To reduce pollution comparatively using less energy, these operational parameters must be optimized. Using turbulence during electro-oxidation might improve oxidation while using less electricity. By adding turbulence during electro-oxidation, the time it takes to reach maximal oxidation can be cut in half.

### 3.5. Effects of Interactions for ECO Combination with UV/H_2_O_2_

**Figure 7:**
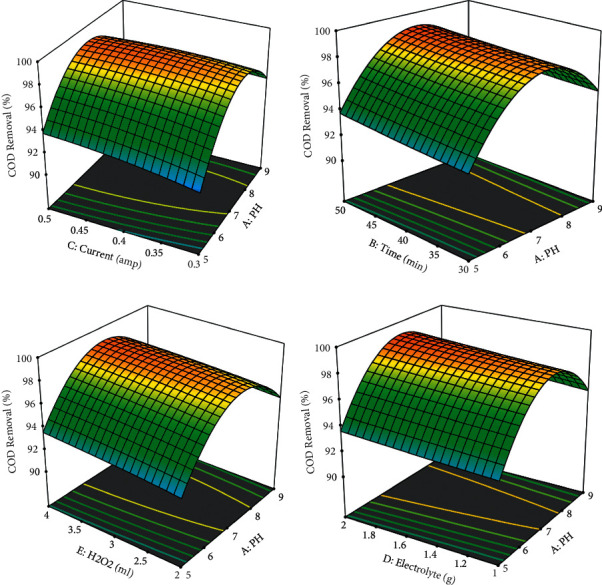
Interaction effect of independent variables 3D response surface plots for % removal of COD, by the combination of ECO with UV/H_2_O_2_ using CaCl_2_.

The effectiveness of pollutant removal in coffee processing wastewater is affected positively or negatively by interactions between more than two independent factors. As shown in the 3D in Figure 7, there are certain interaction effects.

The removal efficiency of COD was high at neutral pH, which is shown by the red color, and when the amount of hydrogen peroxide is 3 mL, as shown in the 3D graph, indicating the interaction impact of pH and hydrogen peroxide favorably influenced the process. Figures 2 and 3 show information on the findings of the interactions between the independent variable and the dependent variable (COD), time, and pH. As shown in Figures 2–6, current in ampere, electrolysis duration, solution pH, and salt content may all have a positive or negative impact on COD removal and power consumption depending on the reaction. As shown in the 3D graphs in Figure 7, increasing the pH from acidic to neutral increases the COD removal rate, whereas decreasing the pH from neutral to basic decreases the COD removal rate decreased. At pH ranges of 7-8, time intervals of 30–50 minutes, current ranges of 0.4–0.6 amperes, and CaCl_2_ concentrations of 1.5–2 g, the highest COD removal rate was identified. When the initial concentration of salt is increased, more hydrogen peroxide is decomposed and the degradation rate increases. Previous research has also found that the greatest catalytic activity for coffee manufacturing wastewater is around pH 6.8 [20]. In addition to this, in the supplementary files Tables S4 and S5, the operating cost for COD removal from coffee processing industrial wastewater was determined. The cost of operation is determined by Ethiopian Birr because the project was performed in Ethiopia. Thus, the cost power is 0.75 cent/KWhr. According to Table S4, at pH (7), time (60 min), current (0.5 amp), electrolyte (1.5 g/L), voltage used for NaCl (2.25 V), power consumed (1.125 KWhr/m^3^), power cost (0.844 ETB/m^3^), and COD removal (94.991%). At pH (7), time (60 min), current (0.5 amp), electrolyte (1.5 g/L), voltage used for CaCl_2_ (2.1911 V), power consumed (1.096 KWhr/m^3^), power cost (0.822 ETB/m^3^), and COD removal (96.387%). As given in Table S5, for H_2_O_2_ (5 ml)/UV used, the maximum COD removed by using NaCl and CaCl_2_ was 98.771% and 99.913%, respectively. The cost of operation at this removal efficiency is 1.350 ETB/m^3^ and 1.130 ETB/m^3^.

### 3.6. Previously Investigated Related Research

Collivignarelli et al. [38] stated that the pollutant from wastewater removals obtained using the UV/H_2_O_2_ oxidation method was close to 90%. The rate of total organic carbon (TOC) degradation increased when the pretreatment techniques were used, indicating that effluent pretreatment is required to improve UV/H_2_O_2_ oxidation performance. The following were discovered to be the best settings for ECO; pH 5, current density 49.1 mA·cm^2^, and operating time 60 minutes. The trials revealed that the ECO procedure removed 75% of COD.

The use of just the right amount of hydrogen peroxide can speed up 4-NP breakdown, but too much hydrogen peroxide slows it down [39]. The use of a sequential EC and UV treatment of tannery effluent has been shown to reduce COD. This method of treatment reduced COD by 94.1%, compared to 85.7 and 55.9% for the solo EC and UV treatments, respectively. A sequential EC and UV treatment of tannery wastewater has been proven effective in the reduction of COD. These treatments reduced COD by 94.1%, whereas the solo EC and UV treatments reduced COD by 85.7 and 55.9%, respectively [15]. The ECO and chemical oxidation processes are both suitable for treating wastewater from the sugarcane sector. At pH 6.5, electrode gap 20 mm, and current density 156 Am^2^, 76% chemical oxygen demand and 79% color removal were obtained with ECO treatment. The addition of a 0.5 M (NaCl) electrolyte concentration improved treatment efficacy by 85% for chemical oxygen demand. Ferrous sulfate and ferric chloride were utilized to increase pollution reduction. At pH 6.5 and 5 mM mass loading, a total COD reduction of 98% and a color reduction of 99.2% were achieved using a combination of ferric chloride [22].

### 3.7. The Prediction of the Optimum Conditions of Responses

The optimal values, the prediction, and experimental findings are in good agreement, indicating that the model is very valid. This initial model's expected *R*^2^ was 99.09%. To build a parsimonious model with meaningful predictors, the backward elimination approach was applied. The anticipated model's coefficient of determination revealed a quadratic link between responses and parameters with a decent regression coefficient. The following ECO and UV/H_2_O_2_ settings were calculated as a realistic optimum using Design Expert 11.1.2.0 software: electrolysis duration of 60 minutes, UV lamp of 50 W, the salt content of 1.5 g/l, and pH of 7. To further evaluate the dependability of the theoretical model prediction, verification tests were conducted under ideal conditions (*n* = 5). The experimental findings for removal efficiencies were extremely similar to the expected values, and the differences were not significant (*p* > 0.05). As a result, the constructed model in this study was shown to be suitable and valid.

The models' competence in predicting the removal of these two pollutants is demonstrated by the good correlations between anticipated and actual COD removal values shown in Figures 2–6. Regression models equation S(E1) and aligned diagrams of the interactive relationships between them and the response variable may be used to illustrate the interactive reaction between four independent factors and dependent variables. Diagnostic diagrams, such as the normal probability distribution diagram of residuals and the diagram of projected values vs. real values, can also be used to assess the model's suitability [40–43].

**Figure 8:**
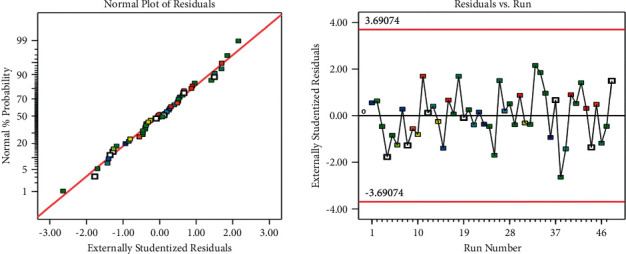
Distribution of normal probability percentage and residuals for COD removal.

The points in these diagrams are all on a pretty straight line, indicating that the variance and normal distribution are both constant. The points are placed along an essentially straight line in the normal probability distribution diagram of residuals. In a normal distribution of data, some of the scattered points are even predicted as shown in Figure 8.

## 4. Conclusions

The response surface methodology (RSM) based on central composite design (CCD) was an effective technique for assessing and optimizing the effects of operational factors on responses. Analysis of variance (ANOVA) with 95% confidence limits was used to examine the significance of independent variables and their interactions. A promising model for predicting chemical oxygen demand (COD) elimination efficiency was presented as the quadratic regression equation SE1. When the greatest removal effectiveness of electrochemical oxidation is combined with UV/H_2_O_2_ utilizing CaCl_2_ for COD removal, the maximum efficiency is 99.7%. The best results were obtained with a pH of 7, a 40-minute electrolysis period, a current of 0.4 Ampere, 1.5 grams of CaCl_2_, and 4 milliliters of H_2_O_2_. This suggests that combining ECO with UV/H_2_O_2_ has a powerful synergistic impact on the elimination of contaminants. CaCl_2_ outperformed NaCl as a supporting electrolyte in both the ECO and UV/H_2_O_2_ processes. ECO in combination with UV/H_2_O_2_ was shown to be a more efficient technology for treating coffee processing wastewater than ECO alone. The maximum COD removal efficiency by using the electrolyte ECO/NaCl and ECO/CaCl_2_ is 94.99 and 96.38, respectively. For H_2_O_2_ with UV combination, the highest COD removal achieved for NaCl was 98.796% and CaCl_2_ was 99.892%.

## Figures and Tables

**Table 1:** CCD results for COD removal by ECO/NaCl and CaCl_2_.

Factors					Responses	
Run	pH	Time (min)	Current (amp)	Electrolyte (g/L)	COD removal efficiency (%) NaCl (g) used	% COD removal by CaCl_2_ (g) used
1	7	60	0.5	1.5	94.991	96.387
2	9	30	0.3	1	91.708	93.204
3	7	40	0.4	2.5	94.951	95.945
4	5	30	0.5	1	89.937	90.333
5	7	40	0.4	1.5	94.241	95.537
6	7	40	0.2	1.5	94.166	95.662
7	5	30	0.3	2	89.566	88.962
8	9	30	0.5	2	92.833	93.329
9	9	50	0.5	2	93.750	94.245
10	7	40	0.4	1.5	94.525	95.175
11	9	40	0.4	1.5	94.083	93.579
12	7	20	0.4	1.5	94.625	95.620
13	5	50	0.3	2	89.916	90.912
14	9	30	0.3	2	92.500	93.095
15	5	50	0.3	1	89.006	89.062
16	7	40	0.4	0.5	92.083	94.579
17	7	60	0.4	1.5	94.925	96.220
18	5	50	0.5	2	90.511	90.995
19	9	50	0.3	2	93.375	94.370
20	5	50	0.5	1	89.291	89.125
21	5	30	0.3	1	88.545	88.041
22	7	40	0.4	1.5	94.629	95.125
23	5	30	0.5	2	89.770	90.966
24	9	50	0.3	1	92.091	93.287
25	7	40	0.6	1.5	94.995	95.791
26	7	40	0.4	1.5	93.051	95.445
27	9	40	0.4	1.5	93.752	93.245
28	7	20	0.4	1.5	93.125	95.620
29	9	30	0.5	1	92.875	94.370
30	9	50	0.5	1	93.841	93.53

**Table 2:** FCCD for COD removal by ECO/NaCl and ECO/CaCl_2_ with UV/H_2_O_2_.

Run	Factor					COD removal efficiency (%)	
	A (pH)	B (time) (min)	C (current) (amp)	D (electrolyte) (g/L)	E (H_2_O_2_) (ml)	NaCl (g) used	CaCl_2_ (g) used
1	5	30	0.3	1	2	91.246	91.904
2	9	30	0.5	2	4	95.783	96.442
3	9	30	0.3	1	4	93.708	94.367
4	5	30	0.5	2	4	92.408	93.067
5	9	50	0.3	2	4	95.025	95.683
6	7	40	0.4	1.5	3	96.683	97.342
7	5	30	0.3	2	2	91.471	92.129
8	9	30	0.5	2	2	93.593	94.251
9	7	60	0.4	1.5	3	98.158	98.817
10	7	40	0.4	1.5	3	97.067	97.725
11	7	40	0.6	1.5	3	98.467	99.125
12	5	30	0.5	2	2	92.575	93.233
13	5	30	0.5	1	4	92.708	93.367
14	7	20	0.4	1.5	3	97.179	97.838
15	5	50	0.3	2	4	91.571	92.229
16	7	40	0.4	1.5	5	98.640	99.298
17	9	30	0.3	2	4	95.438	96.096
18	5	50	0.3	1	4	93.179	93.838
19	7	20	0.5	1.5	3	97.204	97.863
20	9	30	0.3	1	2	94.455	95.113
21	5	50	0.5	1	2	92.575	93.233
22	5	50	0.3	1	2	91.579	92.238
23	5	50	0.3	2	2	91.129	91.788
24	9	50	0.3	1	4	94.458	95.117
25	7	40	0.2	1.5	3	95.063	95.721
26	5	30	0.3	2	4	93.071	93.729
27	5	30	0.5	1	2	92.025	92.683
28	9	30	0.3	2	2	95.354	96.013
29	9	50	0.5	2	2	94.413	95.071
30	7	40	0.4	1.5	3	98.558	99.217
31	7	40	0.4	0.5	3	96.954	97.613
32	9	50	0.5	2	4	95.654	96.313
33	9	30	0.5	1	2	95.079	95.738
34	5	50	0.5	2	2	94.250	94.908
35	9	50	0.3	1	2	95.294	95.952
36	5	30	0.3	1	4	90.294	90.952
37	7	40	0.4	1.5	3	98.379	99.038
38	7	40	0.4	1.5	1	95.096	95.754
39	9	30	0.5	1	4	92.779	93.438
40	7	60	0.4	1.5	3	98.300	99.992
41	9	50	0.5	1	4	95.279	95.938
42	9	50	0.3	2	2	95.813	96.471
43	7	40	0.4	2.5	3	98.796	99.454
44	5	50	0.5	1	4	92.846	93.504
45	7	40	0.4	1.5	3	98.217	98.875
46	9	50	0.5	1	2	93.454	94.113
47	5	50	0.5	2	4	94.213	94.871
48	7	40	0.4	1.5	5	97.771	99.913

**Table 3:** Optimum value of pollutant removed by photoelectrochemical oxidation.

Treatment designs	Major pollutants	Coffee wastewater before treated	Coffee wastewater after treated	Removalefficiency (%)	Permissible WHOstandard for effluents
ECO/NaCl	COD (mg/L)	7680	384.384	94.995	250 mg/l
ECO/CaCl_2_	COD (mg/L)	7680	277.478	96.387	250 mg/l
ECO/NaCl and UV/H_2_O_2_	COD (mg/L)	7680	92.467	98.796	250 mg/l
ECO/CaCl_2_ and UV/H_2_O_2_	COD (mg/L)	7680	8.294	99.892	250 mg/l

## Data Availability

The data used to support the findings of this study are included within the article.

## Supplementary Materials

Supplementary Materials Table S1: ANOVA for the percentage removal of COD by a quadratic model using NaCl. Table S2: ANOVA for percentage removal of COD by the quadratic model using UV/H_2_O_2_ and NaCl. Table S3: model summary (fit statistics) for percentage removal of COD using NaCl. Table S4: CCD results for COD removal by ECO/NaCl and CaCl_2_. Table S5: FCCD for COD removal by ECO/NaCl and ECO/CaCl_2_ with UV/H_2_O_2_. SE1: regression models equation (supplementary equation).
