# Supplementary material for: Wastewater Treatment Using a Photoelectrochemical Oxidation Process for the Coffee Processing Industry Optimization of Chemical Oxygen Demand (COD) Removal Using Response Surface Methodology
Source: Int J Anal Chem. 2022 Jul 31;2022:1734411. doi: 10.1155/2022/1734411 (PMC9357802; doi:10.1155/2022/1734411)
Supplement: Supplementary Materials — Table S1: ANOVA for the percentage removal of COD by a quadratic model using NaCl. Table S2: ANOVA for percentage removal of COD by the quadratic model using UV/H2O2 and NaCl. Table S3: model summary (fit statistics) for percentage removal of COD using NaCl. Table S4: CCD results for COD removal by ECO/NaCl and CaCl2. Table S5: FCCD for COD removal by ECO/NaCl and ECO/CaCl2 with UV/H2O2. SE1: regression models equation (supplementary equation). [file 1734411.f1.docx]

Table S.1: ANOVA for the percentage removal of COD by quadratic model using NaCl

| Source | F-value  (NaCl) | p-value  (NaCl) | F-value  CaCl_2_ | p-value  CaCl_2_ |  |
| --- | --- | --- | --- | --- | --- |
| Model | 21.97 | < 0.0001 | 118.74 | < 0.0001 | significant |
| A-PH | 134.17 | < 0.0001 | 621.18 | < 0.0001 |  |
| B-Time | 4.71 | 0.0464 | 12.03 | 0.0034 |  |
| C-Current | 6.53 | 0.0219 | 16.81 | 0.0009 |  |
| D-Electrolyte | 12.26 | 0.0032 | 29.71 | < 0.0001 |  |
| AB | 0.8093 | 0.3825 | 0.0733 | 0.7902 |  |
| AC | 0.2139 | 0.6503 | 5.07 | 0.0397 |  |
| AD | 0.1756 | 0.6811 | 12.79 | 0.0028 |  |
| BC | 0.0088 | 0.9265 | 17.21 | 0.0009 |  |
| BD | 0.4772 | 0.5003 | 15.54 | 0.0013 |  |
| CD | 1.54 | 0.2334 | 1.48 | 0.2427 |  |
| A² | 138.81 | < 0.0001 | 965.50 | < 0.0001 |  |
| B² | 0.0519 | 0.8229 | 11.41 | 0.0041 |  |
| C² | 0.0162 | 0.9004 | 2.95 | 0.1062 |  |
| D² | 4.02 | 0.0632 | 0.0370 | 0.8501 |  |
| Lack of Fit | 0.5492 | 0.8036 | 3.93 | 0.0721 | not significant |

Table S.2: ANOVA for percentage removal of COD by the quadratic model using UV/H_2_O_2_and NaCl

| Source | F-value  UV/H_2_O_2_/ECO (NaCl) | p-value  UV/H_2_O_2_/ECO (NaCl) | F-value  UV/H_2_O_2_and CaCl_2_ | p-value  UV/H_2_O_2_and CaCl_2_ |  |
| --- | --- | --- | --- | --- | --- |
| Model | 14.07 | < 0.0001 | 13.40 | < 0.0001 | significant |
| A-PH | 52.97 | < 0.0001 | 47.34 | < 0.0001 |  |
| B-Time | 3.83 | 0.0608 | 4.58 | 0.0415 |  |
| C-Current | 5.26 | 0.0299 | 4.59 | 0.0413 |  |
| D-Electrolyte | 6.02 | 0.0209 | 5.38 | 0.0282 |  |
| E-H_2_O_2_ | 4.14 | 0.0519 | 4.97 | 0.0343 |  |
| AB | 0.1969 | 0.6608 | 0.1759 | 0.6782 |  |
| AC | 6.60 | 0.0160 | 5.90 | 0.0221 |  |
| AD | 0.1945 | 0.6627 | 0.1738 | 0.6800 |  |
| AE | 0.2748 | 0.6044 | 0.2456 | 0.6242 |  |
| BC | 0.2535 | 0.6187 | 0.2837 | 0.5986 |  |
| BD | 0.5726 | 0.4558 | 0.5117 | 0.4806 |  |
| BE | 0.3968 | 0.5340 | 0.3546 | 0.5565 |  |
| CD | 0.0791 | 0.7807 | 0.0707 | 0.7924 |  |
| CE | 0.3913 | 0.5369 | 0.3497 | 0.5592 |  |
| DE | 0.9035 | 0.3503 | 0.8075 | 0.3768 |  |
| A² | 131.03 | < 0.0001 | 128.24 | < 0.0001 |  |
| B² | 0.0060 | 0.9388 | 0.1037 | 0.7499 |  |
| C² | 1.69 | 0.2046 | 1.59 | 0.2179 |  |
| D² | 0.0158 | 0.9008 | 0.0120 | 0.9135 |  |
| E² | 1.36 | 0.2537 | 0.2261 | 0.6382 |  |
| Lack of Fit | 1.78 | 0.2442 | 1.72 | 0.2581 | not significant |

Table S.3: Model Summary (Fit Statistics) for percentage removal of COD using NaCl

|  | % COD removals using NaCl | % COD removals using CaCl_2_ | % COD removals using Combination NaCl and UV/H_2_O_2_ | % COD removals using Combination CaCl_2_ and UV/H_2_O_2_ |
| --- | --- | --- | --- | --- |
| Std. Dev. | 0.6214 | 0.3239 | 0.9337 | 0.9876 |
| Mean | 92.59 | 93.39 | 94.86 | 95.57 |
| C.V. % | 0.6712 | 0.3468 | 0.9842 | 1.03 |
| R² | 0.9535 | 0.9911 | 0.9125 | 0.9085 |
| Adjusted R² | 0.9101 | 0.9827 | 0.8476 | 0.8407 |
| Predicted R² | 0.8330 | 0.9465 | 0.6955 | 0.6837 |
| Adeq Precision | 14.6749 | 35.1243 | 12.4325 | 11.8832 |

Table S.4: CCD results for COD removal by ECO/NaCl and CaCl_2_

| Factors | | | | | | | Responses | | | | | |
| --- | --- | --- | --- | --- | --- | --- | --- | --- | --- | --- | --- | --- |
| Run | PH | Time (Min) | Current (amp) | Electrolyte    (g/L) | voltage used  for Nacl | Power consumed KWhr/m^3^ | power cost ETB/m3 | %COD  Removal   using Nacl | voltage used  for Cacl_2_ | Power consumed KWhr/m^3^ | power cost ETB/m^3^  (0.75cent/KWh) | %COD Removal  using Cacl_2_ |
| 1 | 7 | 60 | 0.5 | 1.5 | 2.25 | 1.125 | 0.844 | 94.991 | 2.1911 | 1.096 | 0.822 | 96.387 |
| 2 | 9 | 30 | 0.3 | 1 | 2.35 | 0.353 | 0.265 | 91.708 | 2.2911 | 0.344 | 0.258 | 93.204 |
| 3 | 7 | 40 | 0.4 | 2.5 | 1.85 | 0.493 | 0.370 | 94.951 | 1.7911 | 0.478 | 0.359 | 95.945 |
| 4 | 5 | 30 | 0.5 | 1 | 4.25 | 1.063 | 0.797 | 89.937 | 4.1911 | 1.048 | 0.786 | 90.333 |
| 5 | 7 | 40 | 0.4 | 1.5 | 1.81 | 0.483 | 0.362 | 94.241 | 1.7511 | 0.467 | 0.350 | 95.537 |
| 6 | 7 | 40 | 0.2 | 1.5 | 1.8 | 0.240 | 0.180 | 94.166 | 1.7411 | 0.232 | 0.174 | 95.662 |
| 7 | 5 | 30 | 0.3 | 2 | 1.85 | 0.278 | 0.209 | 89.566 | 1.7911 | 0.269 | 0.202 | 88.962 |
| 8 | 9 | 30 | 0.5 | 2 | 2.55 | 0.638 | 0.479 | 92.833 | 2.4911 | 0.623 | 0.467 | 93.329 |
| 9 | 9 | 50 | 0.5 | 2 | 1.81 | 0.754 | 0.566 | 93.705 | 1.7511 | 0.730 | 0.548 | 94.245 |
| 10 | 7 | 40 | 0.4 | 1.5 | 1.8 | 0.480 | 0.360 | 94.525 | 1.7411 | 0.464 | 0.348 | 95.175 |
| 11 | 9 | 40 | 0.4 | 1.5 | 1.82 | 0.485 | 0.364 | 94.083 | 1.7611 | 0.470 | 0.353 | 93.579 |
| 12 | 7 | 20 | 0.4 | 1.5 | 2.4 | 0.320 | 0.240 | 94.625 | 2.3411 | 0.312 | 0.234 | 95.620 |
| 13 | 5 | 50 | 0.3 | 2 | 1.91 | 0.478 | 0.359 | 89.916 | 1.8511 | 0.463 | 0.347 | 90.912 |
| 14 | 9 | 30 | 0.3 | 2 | 2.7 | 0.405 | 0.304 | 92.511 | 2.6411 | 0.396 | 0.297 | 93.095 |
| 15 | 5 | 50 | 0.3 | 1 | 1.55 | 0.388 | 0.291 | 89.006 | 1.4911 | 0.373 | 0.280 | 89.062 |
| 16 | 7 | 40 | 0.4 | 0.5 | 2.61 | 0.696 | 0.522 | 92.083 | 2.5511 | 0.680 | 0.510 | 94.579 |
| 17 | 7 | 60 | 0.4 | 1.5 | 1.83 | 0.732 | 0.549 | 94.925 | 1.7711 | 0.708 | 0.531 | 96.220 |
| 18 | 5 | 50 | 0.5 | 2 | 2.6 | 1.083 | 0.812 | 90.511 | 2.5411 | 1.059 | 0.794 | 90.995 |
| 19 | 9 | 50 | 0.3 | 2 | 2.62 | 0.655 | 0.491 | 93.375 | 2.5611 | 0.640 | 0.480 | 94.370 |
| 20 | 5 | 50 | 0.5 | 1 | 1.86 | 0.775 | 0.581 | 89.291 | 1.8011 | 0.750 | 0.563 | 89.125 |
| 21 | 5 | 30 | 0.3 | 1 | 1.85 | 0.278 | 0.209 | 88.545 | 1.7911 | 0.269 | 0.202 | 88.041 |
| 22 | 7 | 40 | 0.4 | 1.5 | 1.84 | 0.491 | 0.368 | 94.629 | 1.7811 | 0.475 | 0.356 | 95.125 |
| 23 | 5 | 30 | 0.5 | 2 | 2.75 | 0.688 | 0.516 | 89.77 | 2.6911 | 0.673 | 0.505 | 90.966 |
| 24 | 9 | 50 | 0.3 | 1 | 2.74 | 0.685 | 0.514 | 92.091 | 2.6811 | 0.670 | 0.503 | 93.287 |
| 25 | 7 | 40 | 0.6 | 1.5 | 2.75 | 1.100 | 0.825 | 94.995 | 2.6911 | 1.076 | 0.807 | 95.791 |
| 26 | 7 | 40 | 0.4 | 1.5 | 1.81 | 0.483 | 0.362 | 93.051 | 1.7511 | 0.467 | 0.350 | 95.445 |
| 27 | 9 | 40 | 0.4 | 1.5 | 1.85 | 0.493 | 0.370 | 93.752 | 1.7911 | 0.478 | 0.359 | 93.245 |
| 28 | 7 | 20 | 0.4 | 1.5 | 1.65 | 0.220 | 0.165 | 93.125 | 1.5911 | 0.212 | 0.159 | 95.620 |
| 29 | 9 | 30 | 0.5 | 1 | 2.8 | 0.700 | 0.525 | 92.875 | 2.7411 | 0.685 | 0.514 | 94.370 |
| 30 | 9 | 50 | 0.5 | 1 | 2.81 | 1.171 | 0.878 | 93.841 | 2.7511 | 1.146 | 0.860 | 93.53 |

Table S.5: FCCD for COD removal by ECO/NaCl and ECO/CaCl_2_with UV/H_2_O_2_

| Run | A:  pH | B: Time  (min) | C:Current  (amp) | D: Electrolyte  (g) | E:H_2_O_2_  (ml) | voltage used for CaCl_2_/H_2_O_2_ | power KWhr/m^3^ | P.cost  ETB/m^3^ | %COD  Removal  using Nacl/H_2_O_2_ | voltage used for CaCl_2_/H_2_O_2_ | Power consumed KWhr/m^3^ | p.cost  ETB/m^3^ | %COD  Removal  using H_2_O_2_/Cacl_2_ |
| --- | --- | --- | --- | --- | --- | --- | --- | --- | --- | --- | --- | --- | --- |
| 1 | 5 | 30 | 0.3 | 1 | 2 | 4.5 | 0.675 | 0.506 | 91.246 | 3.920 | 0.588 | 0.441 | 91.904 |
| 2 | 9 | 30 | 0.5 | 2 | 4 | 4.6 | 1.150 | 0.863 | 95.783 | 4.032 | 1.008 | 0.756 | 96.442 |
| 3 | 9 | 30 | 0.3 | 1 | 4 | 3.9 | 0.585 | 0.439 | 93.708 | 3.332 | 0.500 | 0.375 | 94.367 |
| 4 | 5 | 30 | 0.5 | 2 | 4 | 2.8 | 0.700 | 0.525 | 92.408 | 2.232 | 0.558 | 0.419 | 93.067 |
| 5 | 9 | 50 | 0.3 | 2 | 4 | 2.7 | 0.675 | 0.506 | 95.025 | 2.132 | 0.533 | 0.400 | 95.683 |
| 6 | 7 | 40 | 0.4 | 1.5 | 3 | 4.1 | 1.093 | 0.820 | 96.683 | 3.532 | 0.942 | 0.707 | 97.342 |
| 7 | 5 | 30 | 0.3 | 2 | 2 | 4.2 | 0.630 | 0.473 | 91.471 | 3.632 | 0.545 | 0.409 | 92.129 |
| 8 | 9 | 30 | 0.5 | 2 | 2 | 4.8 | 1.200 | 0.900 | 93.593 | 4.232 | 1.058 | 0.794 | 94.251 |
| 9 | 7 | 60 | 0.4 | 1.5 | 3 | 4.9 | 1.960 | 1.470 | 98.158 | 4.332 | 1.733 | 1.300 | 98.817 |
| 10 | 7 | 40 | 0.4 | 1.5 | 3 | 4.5 | 1.200 | 0.900 | 97.067 | 3.921 | 1.046 | 0.785 | 97.725 |
| 11 | 7 | 40 | 0.6 | 1.5 | 3 | 4.5 | 1.800 | 1.350 | 98.467 | 3.932 | 1.573 | 1.180 | 99.125 |
| 12 | 5 | 30 | 0.5 | 2 | 2 | 4.4 | 1.100 | 0.825 | 92.575 | 3.832 | 0.958 | 0.719 | 93.233 |
| 13 | 5 | 30 | 0.5 | 1 | 4 | 4.3 | 1.075 | 0.806 | 92.708 | 3.721 | 0.930 | 0.698 | 93.367 |
| 14 | 7 | 20 | 0.4 | 1.5 | 3 | 2.8 | 0.373 | 0.280 | 97.179 | 2.232 | 0.298 | 0.224 | 97.838 |
| 15 | 5 | 50 | 0.3 | 2 | 4 | 5.8 | 1.450 | 1.088 | 91.571 | 5.232 | 1.308 | 0.981 | 92.229 |
| 16 | 7 | 40 | 0.4 | 1.5 | 5 | 4.4 | 1.173 | 0.880 | 98.64 | 3.832 | 1.022 | 0.767 | 99.298 |
| 17 | 9 | 30 | 0.3 | 2 | 4 | 5.9 | 0.885 | 0.664 | 95.438 | 5.332 | 0.800 | 0.600 | 96.096 |
| 18 | 5 | 50 | 0.3 | 1 | 4 | 6.1 | 1.525 | 1.144 | 93.179 | 5.532 | 1.383 | 1.037 | 93.838 |
| 19 | 7 | 20 | 0.5 | 1.5 | 3 | 4.7 | 0.783 | 0.587 | 97.204 | 4.132 | 0.689 | 0.517 | 97.863 |
| 20 | 9 | 30 | 0.3 | 1 | 2 | 3.2 | 0.480 | 0.360 | 94.455 | 2.632 | 0.395 | 0.296 | 95.113 |
| 21 | 5 | 50 | 0.5 | 1 | 2 | 4.8 | 2.000 | 1.500 | 92.575 | 4.231 | 1.763 | 1.322 | 93.233 |
| 22 | 5 | 50 | 0.3 | 1 | 2 | 4.7 | 1.175 | 0.881 | 91.579 | 4.132 | 1.033 | 0.775 | 92.238 |
| 23 | 5 | 50 | 0.3 | 2 | 2 | 3.8 | 0.950 | 0.713 | 91.129 | 3.232 | 0.808 | 0.606 | 91.788 |
| 24 | 9 | 50 | 0.3 | 1 | 4 | 3.3 | 0.825 | 0.619 | 94.458 | 2.732 | 0.683 | 0.512 | 95.117 |
| 25 | 7 | 40 | 0.2 | 1.5 | 3 | 2.1 | 0.280 | 0.210 | 95.063 | 1.532 | 0.204 | 0.153 | 95.721 |
| 26 | 5 | 30 | 0.3 | 2 | 4 | 4.7 | 0.705 | 0.529 | 93.071 | 4.132 | 0.620 | 0.465 | 93.729 |
| 27 | 5 | 30 | 0.5 | 1 | 2 | 4.2 | 1.050 | 0.788 | 92.025 | 3.632 | 0.908 | 0.681 | 92.683 |
| 28 | 9 | 30 | 0.3 | 2 | 2 | 2.5 | 0.375 | 0.281 | 95.354 | 1.932 | 0.290 | 0.218 | 96.013 |
| 29 | 9 | 50 | 0.5 | 2 | 2 | 2.8 | 1.167 | 0.875 | 94.413 | 2.232 | 0.930 | 0.698 | 95.071 |
| 30 | 7 | 40 | 0.4 | 1.5 | 3 | 2.3 | 0.613 | 0.460 | 98.558 | 1.732 | 0.462 | 0.347 | 99.217 |
| 31 | 7 | 40 | 0.4 | 0.5 | 3 | 2.4 | 0.640 | 0.480 | 96.954 | 1.831 | 1.831 | 1.373 | 97.613 |
| 32 | 9 | 50 | 0.5 | 2 | 4 | 2.8 | 1.167 | 0.875 | 95.654 | 2.232 | 2.232 | 1.674 | 96.313 |
| 33 | 9 | 30 | 0.5 | 1 | 2 | 2.6 | 0.650 | 0.488 | 95.079 | 2.032 | 2.032 | 1.524 | 95.738 |
| 34 | 5 | 50 | 0.5 | 2 | 2 | 4.3 | 1.792 | 1.344 | 94.25 | 3.732 | 3.732 | 2.799 | 94.908 |
| 35 | 9 | 50 | 0.3 | 1 | 2 | 2.1 | 0.525 | 0.394 | 95.294 | 1.532 | 1.532 | 1.149 | 95.952 |
| 36 | 5 | 30 | 0.3 | 1 | 4 | 4.7 | 0.705 | 0.529 | 90.294 | 4.132 | 4.132 | 3.099 | 90.952 |
| 37 | 7 | 40 | 0.4 | 1.5 | 3 | 2.3 | 0.613 | 0.460 | 98.379 | 1.732 | 1.732 | 1.299 | 99.038 |
| 38 | 7 | 40 | 0.4 | 1.5 | 1 | 2.2 | 0.587 | 0.440 | 95.096 | 1.632 | 1.632 | 1.224 | 95.754 |
| 39 | 9 | 30 | 0.5 | 1 | 4 | 3.4 | 0.850 | 0.638 | 92.779 | 2.832 | 2.832 | 2.124 | 93.438 |
| 40 | 7 | 60 | 0.4 | 1.5 | 3 | 2.5 | 1.000 | 0.750 | 98.3 | 1.932 | 1.932 | 1.449 | 99.992 |
| 41 | 9 | 50 | 0.5 | 1 | 4 | 2.9 | 1.208 | 0.906 | 95.279 | 2.332 | 2.332 | 1.749 | 95.938 |
| 42 | 9 | 50 | 0.3 | 2 | 2 | 2.8 | 0.700 | 0.525 | 95.813 | 2.232 | 2.232 | 1.674 | 96.471 |
| 43 | 7 | 40 | 0.4 | 2.5 | 3 | 2.3 | 0.613 | 0.460 | 98.796 | 1.732 | 1.732 | 1.299 | 99.454 |
| 44 | 5 | 50 | 0.5 | 1 | 4 | 3.8 | 1.583 | 1.187 | 92.846 | 3.211 | 3.211 | 2.408 | 93.504 |
| 45 | 7 | 40 | 0.4 | 1.5 | 3 | 2.2 | 0.587 | 0.440 | 98.217 | 1.632 | 1.632 | 1.224 | 98.875 |
| 46 | 9 | 50 | 0.5 | 1 | 2 | 3.1 | 1.292 | 0.969 | 93.454 | 2.532 | 2.532 | 1.899 | 94.113 |
| 47 | 5 | 50 | 0.5 | 2 | 4 | 3.7 | 1.542 | 1.157 | 94.213 | 3.132 | 3.132 | 2.349 | 94.871 |
| 48 | 7 | 40 | 0.4 | 1.5 | 5 | 2.1 | 0.560 | 0.420 | 97.771 | 1.507 | 1.507 | 1.130 | 99.913 |

Rate of power cost =0.75cent/KWh

$\% Removal of COD = 98.44+1.2A+0.3073B+0.3322C+0.3621D+0.3370E-0.0732AB-0.4241AC+0.0728AD-+0.0865AE+0.0895BC-O.1249BD+0.1040BE+0.0464CD+1032CE+1569DE-3.99A^{2}+0.0526B^{2}-0.2572C^{2}+0.0226D^{2}+0.0865E^{2} (SE1)$

SE1: Regression models equation
